# Bioavailability of Tryptophan Biomass for Laying Hens

**DOI:** 10.3390/ani15060866

**Published:** 2025-03-18

**Authors:** Stéphane Cristyne O. Estevão, Gabriel Henrique Nacamura da Silva, Livia Rastoldo R. Oliveira, Larissa Oliveira dos Santos, Erikson Kadoshe M. Raimundo, Rita Brito Vieira, Tiago A. Rodrigues, Bernardo Rocha F. Nogueira, Eliane Aparecida da Silva, Lizandra Amoroso, Michele Bernardino de Lima, Edney Pereira da Silva

**Affiliations:** 1 Laboratory of Poultry Science, Department of Animal Sciences, School of Agricultural and Veterinary Sciences, UNESP-São Paulo State University, Jaboticabal 14884-900, SP, Brazil; s.estevao@unesp.br (S.C.O.E.); gabriel.nacamura@unesp.br (G.H.N.d.S.); livia.r.oliveira@unesp.br (L.R.R.O.); larissa.oliveira-santos@unesp.br (L.O.d.S.); kadoshe.morais@unesp.br (E.K.M.R.); rita.brito@unesp.br (R.B.V.); ta.rodrigues@unesp.br (T.A.R.); lizandra.amoroso@unesp.br (L.A.); 2 CJ Bio Brazil, São Paulo 04571-010, SP, Brazil; bernardo.nogueira@cj.net (B.R.F.N.); eliane.silva@cj.net (E.A.d.S.); 3 Laboratory of Poultry Science, Department of Animal Production and Health, School of Veterinary Medicine, UNESP-São Paulo State University, Araçatuba 16050-680, SP, Brazil; michele.bernardino@unesp.br

**Keywords:** bioequivalence, direct comparison method, egg production, slope ratio method

## Abstract

Two concurrent assays were conducted with the objective of evaluating the bioequivalence and determining the relative bioavailability value (RBV) of L-tryptophan biomass 60% for laying hens. In both assays, L-tryptophan 98% was used as the reference source. A total of 216 hens were used in the two assays. The first assay was performed using the direct comparison method to assess bioequivalence between the sources. In the second assay, the slope ratio method was used to determine the RBV. The variables analyzed were feed intake, tryptophan intake, egg production, egg weight, daily egg mass, feed conversion ratio, feed efficiency, yolk weight, shell weight, albumen weight, albumen height, eggshell thickness, Haugh unit, body protein weight, body lipid weight, body water weight, and body ash weight. The data from both assays were submitted to analysis of variance, and we only applied linear regression to variables from the data obtained using the slope ratio method that were significant at 0.05. The results obtained via the direct comparison method showed no significant difference (*p* > 0.05) between the L-tryptophan sources when using ANOVA. In the slope ratio method, the treatments only affected the egg production and daily tryptophan intake responses of the hens and linear equations were adjusted. No differences (*p* > 0.05) were found for egg quality. When we applied the slope ratio in adjusted equations, we found that the RBV = 100%. The results showed that the utilization of L-tryptophan biomass 60% in the diet of laying hens was bioequivalent.

## Simple Summary

There has been extensive research on amino acid nutrition for layers over the past five decades, but the studies have used a single source of supplemental tryptophan, L-tryptophan 98%. Bioavailability is a crucial aspect of nutrient utilization, encompassing the processes of the digestion, absorption, metabolism, and utilization of the nutrient. This research was conducted to evaluate bioequivalence and determine the relative bioavailability value (RBV) of 60% L-tryptophan biomass for commercial Hy-Line Brown laying hens. Two assays were performed using a completely randomized experimental design. The first assay used a direct comparison method. A total of 66 layers were distributed in two treatments and 11 replicates, with 3 birds per replicate, totaling 21 experimental units. The second assay used the slope ratio method. A total of 150 layers were distributed in such a way that there were five treatments and ten replicates of three hens, totaling 50 experimental units. Performance, egg quality, and body composition were evaluated. The results obtained with the direct comparison method showed no significant difference (*p* > 0.05) between the L-tryptophan biomass 60% and L-tryptophan 98%. Among the body composition variables, only body lipids showed a significant effect (*p* < 0.05). According to the results obtained, the RBV of L-tryptophan biomass 60% was determined at 100% relative to L-tryptophan 98%. Thus, L-tryptophan biomass 60% is bioequivalent to and equally bioavailable as L-tryptophan 98%.

## 1. Introduction

Tryptophan was considered a physiologically essential amino acid by Willcock and Hopkins [1], who observed that the survival and welfare of animals (rats) improved when they received tryptophan supplementation in their diets [1]. Ten years later, tryptophan was also identified as an essential dietary component [2]. In subsequent decades, studies have been carried out on the bioavailability of synthetic compounds in different isomers (D and L) and racemic mixtures (DL) of tryptophan, as well as on the additional radicals introduced into amino or carboxyl groups, such as acetyl-, benzoyl-, methylene, and hydrochloride, among others [3,4,5], which are used as stabilizers of the molecule to support industrial production.

Studies on tryptophan requirements (REQ) for birds began in the 1940s with the White Leghorn [6], and only in the 1950s and the 1960s were some studies carried out with laying hens using practical diets based on corn gluten [7] and corn–soybean meal [8]. According to a review conducted on journals, there was a gap of two decades (1970–1989) in the scientific production of tryptophan for commercial laying hens, ending with the results of [9]. In the last two decades, seven studies have been published on tryptophan REQ [10,11,12,13,14,15,16]; 70% of these studies used white laying hens, and 30% used brown laying hens, corresponding to a total of two publications [12,14]. In general, the reviewed studies on REQ from the past five decades used a single source of supplemental tryptophan, L-tryptophan (98%), explaining the lack of studies on the bioavailability of tryptophan sources during this period.

Recently, alternative amino acid sources have been developed for animal feed [17]. The difference between these sources is the amount of biomass contained in the product, which varies from 25% for threonine [17] and 30% for valine [17] to 40% for tryptophan [17,18]. L-tryptophan biomass is produced through fermentation and granules are formed after steam heating [17]. The biomass, which corresponds to approximately 40%, contains protein, essential amino acids, organic acids, and carbohydrates [17]. Therefore, tests must be conducted to precisely measure the relative bioequivalence of crystalline sources traditionally used in the diet. It is important to study new sources of amino acids so that nutritionists have new options to use in the formulation of poultry feed.

The bioavailability of a new source is a crucial aspect of nutrient utilization, which encompasses digestion, absorption, metabolism, and the utilization of nutrients [19,20,21]. This is because advances in biotechnology are constant, with the development of more efficient strains in the production of L-tryptophan, such as the use of Corynebacterium [17] and Escherichia recently developed by Hou et al. [22].

Therefore, studies evaluating the responses of birds to significant biomass contents and determining the potential of the amino acid biomass for each poultry segment are needed. In this context, recent studies on broiler chickens and pigs have evaluated their growth [17,18]; however, studies on laying hens are required.

Recent studies using only ANOVA have shown that biomass amino acid sources are equivalent to traditional crystalline amino acid sources, confirming the similarity between sources to ensure animal performance [17,18]. Our study hypothesizes that L-tryptophan biomass (60%) has a relative bioavailability (RBV) comparable to traditional crystalline L-tryptophan sources and that its inclusion in the diet of Hy-Line Brown laying hens will not negatively affect egg production, egg quality, and body composition. However, further studies are needed to determine the RBV of L-tryptophan biomass 60%. Therefore, this study aimed to evaluate the effect of RBV on production results, the composition and quality of eggs, and the chemical composition of the bodies of commercial Hy-Line Brown laying hens.

## 2. Materials and Methods

### 2.1. Ethical and Study Approvals

Two studies were performed using laying hens brown at the facility of the São Paulo State University. All experimental procedures were approved by the Ethics Committee for the Use of Animals under protocol number 3340/22.

### 2.2. Assay 1: Direct Comparison Method

#### 2.2.1. Housing, Management and Experimental Design

A total of 66 laying hens (60 weeks old) of the Hy-Line Brown variety, with an average body weight of 1763 ± 0.10 kg and egg production, were housed in experimental facilities (open shed) in a two-tier cage system. The experimental unit consisted of individual cages equipped with linear feeders and nipple drinkers. The hens had free access to water, and feed was provided twice a day, totaling 110 g per hen. The experiment lasted 119 days, with an additional 15-day adaptation period, totaling 133 days. The lighting program used during the experiment consisted of 16 h of fluorescent light at an intensity of 30 lux, measured using a lux meter. The average temperature was 23.1 ± 2.0 °C. The experimental design was completely randomized, with two treatments and 11 replicates of three hens, totaling 22 experimental units. Daily management was performed according to the brown Hy-Line guidelines.

#### 2.2.2. Treatments

The amino acid and energy contents of corn and soybean meal were determined using near-infrared spectroscopy and adjusted in the formulations of the experimental diets. Treatments 1 and 2 were formulated to contain 0.172% digestible tryptophan and met the ideal ratio of tryptophan to lysine of 23% [23]. Treatment 1 consisted of supplementation with 1.0 g/kg of L-tryptophan biomass 60%. Treatment 2 involved supplementation with 0.612 g/kg L-tryptophan (98%). The experimental diets were formulated according to nutritional recommendations of [23]. The chemical compositions of the diets are shown in Table S1.

### 2.3. Assay 2: Slope Ratio Method

#### 2.3.1. Animals, Experimental Conditions, and Environment

At 59 weeks of age, 150 Hy-Line Brown laying hens were selected based on their body weight (1730 ± 0.09 kg) and egg production. For maximum uniformity, egg production in each experimental unit was monitored two weeks before the beginning of the study. Thereafter, the hens (61 weeks old) were housed in experimental facilities in metabolic cages (45 L cm × 50 W cm × 45 H cm), with stocking density (750 cm^2^/hen), in a climate-controlled chamber set at 22 °C and 55% humidity. Each cage was equipped with individual feeders and nipple drinkers. The experimental design was completely randomized, with five treatments and ten replicates of three hens, totaling 50 experimental units. The lighting and daily management were the same as in Assay 1. The experiment lasted for 10 weeks.

#### 2.3.2. Treatments and Diets

Treatments consisted of five experimental diets in mash form. The basal diet (**BD**; Treatment 1—**T1**) was formulated to contain 0.125% digestible tryptophan without crystalline L-tryptophan supplementation (Table 1). Treatments 2 and 3 (**T2** and **T3**) were formulated to contain 0.145% and 0.165% digestible tryptophan (supplemented with L-tryptophan 98%, **L-TRP 98%**), respectively, whereas treatments 4 and 5 (**T4** and **T5**) were formulated to contain 0.145% and 0.165% digestible tryptophan (supplemented with L-tryptophan biomass 60%, **L-TRP 60%**), respectively. The levels of tryptophan in the diet (0.00, 0.145, and 0.165%) were used to obtain a response curve to define the bioequivalence of different dosages of L-tryptophan (Table 2).

The ingredients, except for inert and L-tryptophan, were premixed for 5 min to prepare the BD and BD + L-tryptophan treatments. L-tryptophan and inert were added in appropriate proportions (Table 2) to the premixed solution to form the treatments and remixed for 5 min in a horizontal mixer. Only inert was added to treatment 1 (BD).

The BD was made prior to the ones containing L-tryptophan to avoid cross-contamination. Diets did not contain any enzymes, antibiotics, or growth promoters. The nutritional level (Table 3) of experimental diets was formulated according to [23].

### 2.4. Performance

The same procedures—direct comparison method and slope ratio method—were used in both assays to collect data. The hens were fed 110 g of feed daily at the same time each morning. At the end of each week, the leftovers were weighed to quantify weekly feed intake. Body weights were measured at the beginning and end of the assay. Egg production and mortality were recorded daily, and egg weight was measured weekly for three consecutive days. Egg mass was calculated as the average egg weight multiplied by egg production for each treatment in each period, divided by 100. The weekly leftovers per replicate were weighed to calculate feed intake and feed conversion ratio per egg mass. We corrected the feed conversion based on mortality. The variables analyzed were feed intake (**FI**, g/day), body weight (**BW**, g), egg production (**EP**, %), egg weight (**EW**, g), egg mass (**EM**, g/hen/day), and feed conversion ratio (**FCR, g/g**) by egg mass, corrected for mortality.

### 2.5. Egg Assessment

The same procedures—direct comparison method and slope ratio method—were used in both assays to collect data. Shell-less eggs, those with soft shells, and dirty eggs were recorded daily. At the end of the assay, 2 eggs per experimental unit, totaling 22 eggs per treatment, were randomly selected for egg quality analysis. The eggs were evaluated for weight, albumen height, Haugh unit, yolk weight, and eggshell thickness. The eggs were weighed and broken, and the yolk was separated from the albumen and shell. The shells were washed and air-dried for 48 h, and after weighing them, their eggshells’ thickness was measured. Albumen weight was defined as the difference between the egg’s weight and the sum of the yolk and shell weights. Egg, yolk, and eggshell weights were determined using an analytical balance. The eggshell thickness and albumen height were measured using Vernier calipers. The Haugh unit was calculated as previously described [24].

### 2.6. Body Composition

Using only the direct comparison method, a hen with a body weight matching the average of each treatment replicate was selected for body composition analysis using dual-energy X-ray absorptiometry (DEXA, Hologic-QDR^®^ model 13.4.2., Bedford, MA, USA), totaling 50 animals. At the end of the assay, the hens were subjected to an 8 h solid fast to empty the digestive tract, and then they were weighed and sacrificed by cervical dislocation for the analysis of body composition. The total mass, bone mineral content, bone mineral density, fat mass, and lean mass were determined using DEXA; then, the protein, lipid, water, and ash weights were obtained based on the equations of Alves et al. [25]. The calculations were realized as follows: deposition = body weight × protein or lipid or water or ash content.

### 2.7. Chemical Analysis

The amino acid contents of corn and soybean meal were determined using near-infrared spectroscopy and adjusted in the formulations of the experimental diets. The total nitrogen in the diets was determined using the Kjeldahl method, with a factor of 6.25 used for conversion into protein.

### 2.8. Statistical Analysis

In both assays, the data were tabulated and analyzed for assumptions of the homoscedasticity of variance (Brown–Forsythe test) and normality of errors (Cramér–von Mises test) using a mixed model. The experimental unit was considered a random effect, and the treatments were considered a fixed effect. The F-test was used for ANOVA, and regression analysis was conducted for the slope ratio method assay when the null hypothesis was rejected (*p* ≤ 0.05 level of significance).

The RBV of L-tryptophan 60% was estimated using slope ratio analysis, as described by [21], according to the linear model ξ = α + βsXs + βtXt ± ě, where ξ is the value observed; α is the intercept; ě is random error; βs and βt are the slopes for L-tryptophan 98% and L-tryptophan biomass 60%, respectively; and Xs and Xt are the concentrations of L-tryptophan 98% and L-tryptophan biomass 60% in the diet, respectively. For the nonlinear model, ξ = α + δ × (1 − *e*^(βsXs + βtXt)^) ± ě, where ξ, α, ě, βs, βt, Xs and Xt are defined.

The NLMixed model procedure was used for random-effect linear and nonlinear regression analyses. The α was randomized in both models. The r-squared value was obtained from the ratio between the sq model and sq treatment. The asymptote value is α + δ. Statistical analyses were performed using SAS 9.4 (Statistical Analysis for Windows; SAS Institute Inc., Cary, NC, USA).

## 3. Results

Assays were performed, and the results obtained using the applied methods, direct comparison, and the slope ratio are presented below.

### 3.1. Assay 1: Direct Comparison Between L-Tryptophan Biomass 60% and L-Tryptophan 98%

An assessment of 60% L-tryptophan biomass was performed, focusing on the productive performance responses of brown laying hens. These variables were used to evaluate the bioequivalence of L-tryptophan sources over 17 weeks in hens fed a non-limiting diet. Direct comparison was performed between the two sources of L-tryptophan (60% L-tryptophan biomass and 98% L-tryptophan) using a control diet containing 0.172% tryptophan (Table 4). This comparison revealed a numerical superiority of 2.2% in egg production (EP) and 3.7% in egg mass for the diet supplemented with L-tryptophan biomass 60%, although these differences were not statistically significant (*p* > 0.05).

Additional analyses revealed no differences (*p* > 0.05) in daily feed intake, tryptophan intake, egg weight, feed conversion ratio, feed efficiency, change body weight, body weight, body water, body protein, body ash, yolk weight, eggshell weigh, albumen weight, eggshell thickness, Haugh unit and yolk, eggshell, and albumen percentage variables among laying hens fed the same nutritional levels, L-tryptophan biomass 60%, and L-tryptophan 98% (Table 4). Among the body composition variables (Table 4), only body lipids showed a significant effect (*p* < 0.05); hens fed L-tryptophan 60% had 14.8 g more body fat. This corresponded to an increase of 8.6% compared to the level in hens fed L-tryptophan 98%.

### 3.2. Assay 2: Slope Ratio Method to Determine the RBV of L-Tryptophan Biomass 60%

Initially, the data were analyzed using the F-test, and the results showed no significant differences (*p* > 0.05) between the treatments for all of the variables analyzed, as shown in Table 5 and Table 6.

For egg quality (Table 6), the eggshell weight was 0.23 g higher than that found for the BD treatment, corresponding to 3.8% improvement. However, it was not significant (*p* = 0.1256) based on the BD (T1—0.125% L-Trp) compared to that in the other treatments.

The treatments only affected the daily EP and daily tryptophan intake responses of the hens and allowed the fitting of the linear equations (Table 7). In the treatment without the supplementation of L-tryptophan sources (level at 0.125%—L-tryptophan), the daily EP reached was 86.6% with a daily tryptophan intake of 134 mg/hen.

At a level of 0.145%, laying hens ingested 21.5 mg/hen of daily intake of supplemental tryptophan, corrected by the source, with L-tryptophan 98%, and 21.7 mg/hen of daily intake of supplemental tryptophan, with L-tryptophan biomass 60%. EP reached 90.7% with L-tryptophan biomass 98% and 92.0% using L-tryptophan biomass 60%. Similarly, at a nutritional level of 0.165%, the daily intake of supplemental tryptophan, corrected by the source, was 43.7 mg/hen with L-tryptophan 98% and 43.5 mg/hen with L-tryptophan biomass 60%, while EP achieved 92.3% and 92.01%, respectively. It is noteworthy that the daily intake of tryptophan increased linearly with an increase in the nutritional supplementation levels of both sources of L-tryptophan.

A simple two-group model was used to test regression curves. The regression of the EP variable on TrpSup was assumed (Table 7). The variables were measured in hens, grouped according to the L-tryptophan source. We sought to test two hypotheses to answer this question:

(a) Whether sources of L-tryptophan have separate regression curves;
(b) Whether there is a difference in regression slopes for L-tryptophan sources.

The results of the analyses of the intercept and slope hypotheses provide insights into the relationships between these variables. The intercept (90.2°) represents the point where the equation line crosses the vertical axis (EP) when the supplemental tryptophan (TrpSup, horizontal axis) is zero. However, an estimated difference of −5.44 for the intercept between the BD (84.8) and L-tryptophan sources was observed. This difference was significant (*p* = 0.025), suggesting differences in EP in the absence of tryptophan supplementation. The interaction between the slope (3.18) of the equation and the source of L-tryptophan indicates whether there are variations in the responses to EP according to the different sources of L-tryptophan in the diet. This study showed no significant interaction (*p* = 0.614), indicating that the relationship between tryptophan intake and EP did not differ significantly between L-tryptophan sources. The hypothesis that the dependent variable, EP, is explained by a single model when TrpSup changes was accepted.

This assay was performed to determine the RBV using the slope ratio method with L-tryptophan biomass 60% relative to the source L-tryptophan 98% for brown laying hens. The relationship between the daily intake of supplemental tryptophan corrected for source concentration and the EP for each tryptophan source resulted in the equations shown in Table 8.

Two models were fitted to interpret the relationship between supplemental tryptophan intake and daily EP. The estimated parameters are presented in Table 8. For the linear model (EP = 86.3 + 0.153Xs + 0.151Xt, AIC = 247.5), the RBV was calculated as the relationship between the slopes 0.151 ÷ 0.153 = 98.5%. Considering a 95% confidence interval, the lower and upper limits were calculated at 98% and 100%, respectively, resulting in an overall mean of 99%. For the nonlinear model (EP = 84.8 + 8.3(1 − e((−0.055Xs − 0.055Xt))), AIC = 273.4), the RBV was calculated as the relationship between the slopes 0.0553 ÷ 0.0553 = 100%. Considering a 95% confidence interval, the lower and upper limits were calculated at 101% and 107%, respectively, resulting in an overall mean of 103%.

**Figure 1:**
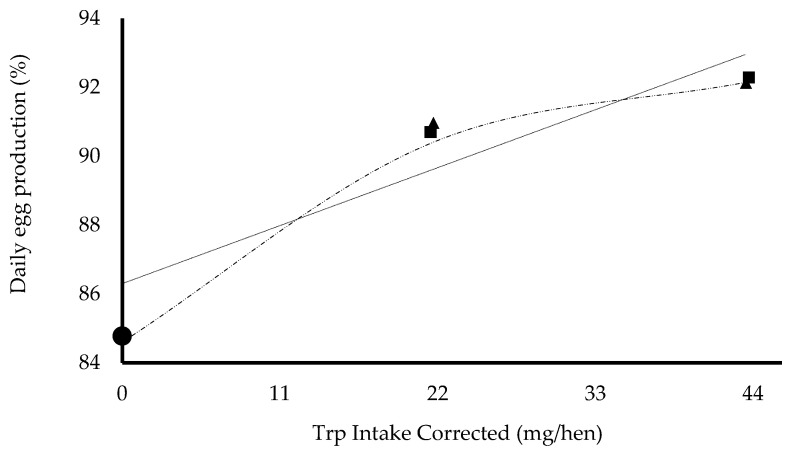
The relationship between daily tryptophan intake supplementation, corrected for source concentration (Trp Intake Corrected) and daily egg production. The bioavailability of L-tryptophan biomass 60% compared to L-tryptophan 98% based on egg production, presented using slope ratio linear (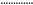) and nonlinear models (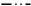). 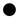, basal diet; 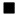, L-tryptophan 98%; 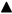, L-tryptophan biomass 60%.

Figure 1 shows the lines predicted by each model and the observed treatment means. Differences were observed between the models in relation to the origin of the predicted curve (Figure 1). The nonlinear model coincided with the average response to the BD (84.8%).

## 4. Discussion

This study was conducted to evaluate whether the L-tryptophan biomass 60% source is bioequivalent to L-tryptophan 98% and to determine the bioavailability of tryptophan in the L-tryptophan biomass 60% source. Two assays were conducted, and the data obtained allowed for statistical analysis and the extraction of the results necessary to answer questions about the bioequivalence and bioavailability of L-tryptophan biomass 60%.

The results obtained using the direct comparison method (Table 4) on tryptophan sources support the hypothesis that the sources are bioequivalent because of the similarities in the responses of laying hens. A direct comparison between tryptophan sources, using a control diet with 0.172% tryptophan (Table 4), revealed a slight numerical superiority of 2.2% in EP and 3.7% in egg mass for the diet supplemented with granular L-tryptophan, although this difference was not statistically significant (*p* > 0.05). A detailed analysis of the responses of laying hens rules out the hypothesis of the mobilization of body reserves to sustain EP, given the improvement observed in production.

A previous study conducted using this method showed that 60 sources of L-tryptophan biomass and L-tryptophan 98% were bioequivalent in terms of ensuring the zootechnical indices of broilers [18]. Also, in research by Lee et al. [18], although not significant at the limit of the margin of error, there is an indication of the better utilization of dietary nitrogen with the supplementation of L-tryptophan biomass 60%. The results obtained in the present study for protein deposition in the bodies of laying hens did not differ between tryptophan sources, which was similar to the findings of Lee et al. [18]. However, nitrogen use in birds directly interferes with lipid metabolism [26] and its utilization rate [27]. In the direct comparison test, the only significant difference (*p* < 0.0001) found between tryptophan sources was an increase in lipid deposition in the bodies of laying hens.

Greater lipid deposition in the body provides energy storage and depends directly on the energy expenditure of the cells demanded by protein metabolism, especially when there is excess nitrogen in the diet [22]. Thus, the hypothesis of excess nitrogen originating from biomass was not supported by the results (Table 4). If this were the case, the cell would demand a greater energy supply to excrete excess nitrogen from the body, and consequently, less energy would be available for lipid synthesis [28,29]. Therefore, there was an energy-saving effect with the use of the source L-tryptophan biomass 60%, but it was not enough to affect feed efficiency owing to its limited inclusion in the diet. The cumulative effect of daily intake of additional calories may have contributed to increased lipid deposition in the bodies of laying hens. In this sense, it is not possible to rule out the hypothesis that the energy value of the L-tryptophan biomass 60% source was underestimated. The metabolizable energy value of 4.288 kcal/kg of the L-tryptophan biomass 60% source was obtained using equation [23]. Therefore, additional studies are needed to confirm the findings of this research and to measure the real supporting contribution of biomass to the animal response.

A combination of methods allows for a more comprehensive and reliable assessment of nutrient availability and provides a solid basis for developing diets that optimize bird performance and health. In the second assay, the RBV was determined using the slope ratio method, and the condition of this method was the relationship between the dose and the hen’s response to ensure the accuracy and reliability of the RBV [21]. Within the sample space defined in this study (Table 5 and Table 6), only EP and tryptophan intake were significant, which allowed the application of the procedure to determine the RBV of tryptophan.

The other variables were not affected by the treatment. Therefore, 0.125% tryptophan in the diet or 134 mg/hen day (Table 7), which corresponds to a reduction of approximately 18% in relation to the recommendation (163 mg/day) of the guidelines, was not sufficient to trigger signs of deficiency in the other variables for an experimental period of 10 weeks, making it impossible to use these variables to determine the RBV.

Egg production was the variable most sensitive to tryptophan levels, as the dose of 0.125% tryptophan in the diet reduced its value after 10 weeks. This result supports the finding of [30], who described how the main response is severely affected by the restriction of amino acid intake, while egg weight rarely decreases. Its greatest reduction has been close to 10%, regardless of how inadequate the amino acid intake is [30,31,32]. Therefore, future studies should use a greater degree of deficiency over a longer period to observe the effects of tryptophan deficiency on other variables, especially those linked to egg quality.

The adaptation period is a problem in research with hens. When the degree of deficiency is small, these hens mobilize body reserves to maintain reproduction, according to previous studies [31,33]. In this research, the 10-week period is traditionally used [31,32,33,34,35]. Among the available information, the tryptophan recommendations ranged from 186 mg/day [23] to 249 mg/day [14], generating a degree of deficiency between 28% and 46%, respectively. However, the results obtained suggest that the requirement for tryptophan is less than 186 mg/day; consequently, the degree of deficiency was less than 28%. This justifies the reduction of only 6 percent units in egg production, which was the result obtained for the basal diet in the evaluated period; therefore, future studies should consider a period longer than 10 weeks to observe effects on the other variables, especially egg quality.

The relationship between the daily intake of supplemental tryptophan, corrected for source concentration, and the EP for each tryptophan source resulted in the equations shown in Table 8. The RBV was determined using linear and nonlinear models, and the results obtained were 99% and 103% of RBV, respectively, for L-tryptophan biomass 60%, relative to L-tryptophan 98%, resulting in an overall mean of 101%. However, the selection of the model to conclude the recommendation was based on the lowest AIC value; therefore, following this criterion, the RBV was equal to 100% for L-tryptophan biomass 60%. This value is the result of the similarity found in the adjusted values for the slope (βt and βs) of the tryptophan sources (0.055 ÷ 0.055 = 1).

Analyzing the quality of the adjustment following the recommendation of Silva et al. [36], the errors associated with the mean value of βt and βs were more significant than those found for the linear adjustment approximately by 2.4 times, and this may have affected the estimation of the confidence interval, which recommends a lower limit very close to the mean value found. Other studies on bioavailability in laying hens have also emphasized the importance of errors in estimating confidence intervals [37,38].

The importance of errors in estimating the confidence interval was corroborated by the values obtained using the linear model. In this model, the errors were smaller, and the confidence interval was symmetrical to the determined mean value of the RBV. A linear model was not selected due to its adjustment to the hen’s responses. From Figure 1, it can be verified that the adjustment of the exponential curve to the observed means is better, regardless of the adjustment and selection statistics. The greater number of parameters in the exponential model allowed for improved interpretation of the hens’ responses, accurately estimating the response to the BD and the maximum response of the hens. These qualities were established by Littell et al. [21] as the criteria that must be met in the bioavailability assay.

According to [21], three assumptions must be made to ensure the validity of the slope ratio assay when using a linear model. The first is to assume linearity in the dose–response relationship, the second is to assume the same intercept between the fitted lines, and the third is to assume that the intercept of the line coincides with the response of the BD. Only one assumption was met by the linear model.

Considering the instantaneous derivative (ξn-ξn-1/Xn-Xn-1), the values between the treatments at 0.145% and 0.125% and at 0.165 and 0.145% were 0.280 and 0.068 production unit per unit of supplemental tryptophan intake (Figure 1), respectively. Therefore, the unitary response rate decreases abruptly by approximately four times between the levels of 0.165% and 0.145%, and the derivative should be approximately constant to be linear. The intercept of the supplement levels is the same between sources, according to Eq.: EP = 90.2 + 3.18TripSup), but it does not coincide with the BD. For this reason, the exponential model (EP = 84.8 + 8.3(1 − e((−0.055Xs − 0.055Xt))), AIC = 273.4) was preferred to assess whether the RBV of the L-tryptophan biomass was 60%. This research confirms the position of previous studies that recommended the careful evaluation of the mathematical model used to establish the RBV [37,38].

It is important to consider the physical form of supplemental amino acids and the digestion and absorption processes of their units. The powdered physical form is most commonly used in animal feed. This form is absorbed more quickly, and for situations that require immediate availability, its use is advantageous to some extent. The physical form of tryptophan in the L-tryptophan biomass source 60% was granules and it has the characteristic of slower release, causing its release to be prolonged.

For situations that require slower release, it is, to some extent, advantageous. This is valid for hens that are normally raised with feeding controls [34]. The results obtained by [35] clearly demonstrated that the higher release speed of synthetic amino acids, and consequently, rapid absorption, can affect the egg mass production response because of the higher concentration of synthetic amino acids in the flow between the intestine, blood, and tissues available for protein synthesis. When [35] increased the number of meals, there was an improvement in egg mass production and, consequently, in the efficiency of the use of supplementary amino acids. Therefore, the physical form of tryptophan granules present in the 60% L-tryptophan biomass source may be an interesting characteristic of these hens.

However, at this time, we only had one hypothesis since the speed of tryptophan passage from different sources in the gastrointestinal tracts of birds was not measured. Thus, the slower release of tryptophan in the 60% L-tryptophan biomass source was limited, supporting an explanation for the upper limit of the confidence interval, which was 107% in relation to the 98% L-tryptophan source. In this study, 100% of the 60% L-tryptophan biomass was considered digestible; therefore, the nutritional matrix assumed a concentration of 60% L-tryptophan to be fully digestible. Although L-tryptophan excretion was not measured, its bioavailability was close to 100%; therefore, excretion was not expected to be affected. This hypothesis was supported by the responses of the birds, considering that there was no reduction in the responses of laying hens to treatments with L-tryptophan biomass 60%.

## 5. Conclusions

No differences were found for egg quality. For body composition, only body lipids showed a significant effect. The bioavailability of L-tryptophan biomass 60% was determined at 100% and it presented the same bioequivalence as the crystalline source of L-tryptophan 98%.

## Figures and Tables

**Table 1:** Composition (kg/ton) of the basal diet used in the assay.

Ingredients	Basal Diet
Grain corn	699.701
Soybean meal 46 of CP% ^1^	146.225
Meat and bone meal 42 of CP% ^1^	49.222
Coarse limestone	15.193
NaCl	75.393
Sodium bicarbonate	3.086
L-Met 100	2.371
L-Lysine	1.753
L-Threonine	0.648
L-Valine	0.376
L-Isoleucine	0.781
Choline chloride 60% of ChoCl ^2^	0.750
Inert (Kaolin)	2.501
Premix ^3^	2.000
Total	1000

^1^ Crude protein; ^2^ choline chloride; ^3^ content per kg of diet: folic acid—1.232 mg; pantothenic acid—26.40 mg; biotin—0.0352 mg; niacin—61.60 mg; vitamin A—4.224 mg; vitamin B1—3.872 mg; vitamin B12—0.02112 mg; vitamin B2—10.032 mg; vitamin B6—5.632 mg; vitamin D3—0.1232 mg; vitamin E—23.584 mg; vitamin K3—3.52 mg; copper—16.00 mg; iron—100.00 mg; iodine—2.40 mg; manganese—140.00 mg; selenium—0.60 mg; zinc—140.00 mg.

**Table 2:** Treatment’s description used in the assay.

Items	T1	T2	T3	T4	T5
	BD ^1^	L-TRP 98% ^2^	L-TRP 98% ^2^	L-TRP 60% ^3^	L-TRP 60% ^3^
Basal	997.499	997.499	997.499	997.499	997.499
Inert (Kaolin)	2.501	2.297	2.093	2.193	1.886
L-Tryptophan 98%	-	0.204	0.408	-	-
L-Tryptophan biomass 60%	-	-	-	0.308	0.615
Total	1000.000	1000.000	1000.000	1000.000	1000.000

^1^ Basal diet; ^2^ L-Tryptophan 98%; ^3^ L-Tryptophan biomass 60%.

**Table 3:** Calculated and analyzed compositions of the experimental diets.

Items	T1	T2	T3	T4	T5
	BD ^1^	L-TRP 98% ^2^	L-TRP 98% ^2^	L-TRP 60% ^3^	L-TRP 60 ^3^
Calculated					
crude protein, %	15.2	15.2	15.2	15.2	15.2
Metabolizable energy, kcal/kg	2800	2801	2803	2801	2803
Lysine, %	0.730	0.730	0.730	0.730	0.730
Methionine + cysteine, %	0.660	0.660	0.660	0.660	0.660
Threonine, %	0.510	0.510	0.510	0.510	0.510
Tryptophan, %	0.125	0.145	0.165	0.145	0.165
Arginine, %	0.805	0.805	0.805	0.806	0.806
Valine, %	0.640	0.640	0.640	0.640	0.640
Isoleucine, %	0.580	0.580	0.580	0.580	0.580
Potassium, %	0.519	0.519	0.519	0.519	0.519
Sodium, %	0.160	0.160	0.160	0.160	0.160
Calcium, %	4.180	4.180	4.180	4.180	4.180
Available phosphorus, %	0.360	0.360	0.360	0.360	0.360
Total choline, mg/kg	1260	1260	1260	1260	1260
Linoleic acid, %	1463	1464	1464	1464	1464
Analyzed					
crude protein, %	15.88	15.94	15.95	16.06	16.07
Tryptophan ^4^, %	0.126	0.146	0.166	0.146	0.166

^1^ Basal diet; ^2^ L-Tryptophan 98%; ^3^ L-Tryptophan biomass 60%; ^4^ total tryptophan × digestibility coefficient from Rostagno et al. [23].

**Table 4:** Average responses to performance, body composition, and egg quality from brown laying hens fed with two non-limiting diets formulated with two sources of L-tryptophan.

Variables	TRP 60% ^1^	TRP 98% ^2^	Average	Stderr ^3^	*p*-Value	CV, % ^4^
Feed intake, g/day hen	108.7	109.2	109.0	0.2	0.125	0.7
Tryptophan intake mg/day hen	186.9	187.9	187.4	0.3	0.125	0.7
Egg production, %	94.1	92.5	93.3	0.5	0.128	2.7
Egg weight, g	59.8	59.4	59.6	0.3	0.447	2.5
Egg mass, g/day hen	56.3	54.9	55.6	0.5	0.115	3.8
Feed conversion ratio g/g	1.932	1.990	1.961	0.017	0.085	4.1
Feed efficiency, g/kg	518.5	502.9	510.7	4.5	0.083	4.1
Change body weight, g/day hen	1.3	1.0	1.2	0.1	0.181	55.8
Body weight, kg	1.9	1.9	1.9	0.0	0.531	4.8
Body water, g	917.3	938.6	928.0	6.6	0.105	3.2
Body protein, g	272.5	278.8	275.6	1.9	0.106	3.2
Body lipid, g	186.7	171.9	179.3	2.1	<0.0001	5.2
Body ash, g	56.7	58.1	57.4	0.4	0.098	3.2
Yolk weight, g	15.5	15.1	15.3	0.1	0.198	3.7
Eggshell weigh, g	5.9	6.0	6.0	0.1	0.650	4.6
Albumen weight, g	38.4	38.2	38.3	0.3	0.767	3.3
Eggshell thickness, µm	398.9	405.3	402.1	3.0	0.290	3.4
Haugh unit, %	95.2	95.8	95.5	0.7	0.677	3.6
Yolk percentage, %	25.9	25.5	25.7	0.2	0.300	3.2
Shell percentage, %	9.9	10.1	10.0	0.1	0.420	4.4
Albumen percentage, %	64.2	64.4	64.3	0.2	0.706	1.6

^1^ L-Tryptophan biomass 60%; ^2^ L-Tryptophan 98%; ^3^ Stderr: standard error of mean; ^4^ coefficient of variation.

**Table 5:** Average responses to treatments for daily feed intake (FI, g/hen), egg weight (EW, g), daily egg mass (EM, g/day), feed conversion ratio (FCR, g/g), and feed efficiency (FE, g/kg) of brown laying hens from 61 to 70 weeks of age.

Treatments	L-Trp ^4^	FI	EW	EM	FCR	FE
T1—BD ^1^	0.125	107.2	60.0	53.0	2.04	493.0
T2—TRP 98% ^2^	0.145	107.7	60.7	55.0	1.96	511.0
T3—TRP 98% ^2^	0.165	109.2	60.0	55.5	1.97	509.0
T4—TRP 60% ^3^	0.145	108.9	59.4	54.6	1.99	503.0
T5—TRP 60% ^3^	0.165	108.7	61.5	56.8	1.93	520.0
Average		108.3	60.3	55.0	1.98	0.507
Stderr ^5^		0.3	0.3	0.5	0.02	0.004
*p*-value		0.182	0.365	0.160	0.390	0.339
CV, % ^6^		0.193	3.68	5.81	5.57	5.53

^1^ Basal diet; ^2^ L-Tryptophan 98%; ^3^ L-Tryptophan biomass 60%; ^4^ level of L-tryptophan; ^5^ standard error of mean; ^6^ coefficient of variation.

**Table 6:** Average responses to treatments in terms of yolk weight (YW, g), shell weight (SW, g), albumen weight (AW, g), albumen height (AH, mm), eggshell thickness (EST, μm), and Haugh unit (HU, %) of brown laying hens from 61 to 70 weeks of age.

Treatments	L-Trp ^4^	YW	SW	AW	AH	EST	HU
T1—BD ^1^	0.125	15.1	5.9	38.0	9.7	406.8	98.1
T2—TRP 98% ^2^	0.145	15.1	6.2	38.3	9.9	403.6	99.3
T3—TRP 98% ^2^	0.165	14.9	6.0	38.7	9.7	398.0	97.7
T4—TRP 60% ^3^	0.145	14.8	6.2	39.0	9.9	411.8	97.5
T5—TRP 60% ^3^	0.165	15.0	6.1	39.2	10.1	408.7	99.4
Average		15.0	6.1	38.6	9.9	405.8	98.4
Stderr ^5^		0.1	0.04	0.3	0.1	2.2	0.4
*p*-value		0.765	0.129	0.599	0.607	0.332	0.491
CV, % ^6^		3.27	3.95	4.37	6.05	3.75	3.05

^1^ Basal diet; ^2^ L-Tryptophan 98%; ^3^ L-Tryptophan biomass 60%; ^4^ level of L-tryptophan; ^5^ standard error of mean; ^6^ coefficient of variation.

**Table 7:** Average responses of daily tryptophan supplementary intake (Trp Intake, mg/hen), daily intake of tryptophan supplemental corrected for source concentration (Trp Intake Corrected, mg/hen) and daily egg production (EP, %) of brown laying hens from 61 to 70 weeks of age.

Treatments	L-Trp ^4^	Trp Sup ^5^	Trp Intake	Trp Intake Corrected	EP
T1—BD ^1^	0.125	0	134.0	0	84.8
T2—TRP 98% ^2^	0.145	0.204	156.2	21.5	90.7
T3—TRP 98% ^2^	0.165	0.408	180.2	43.7	92.3
T4—TRP 60% ^3^	0.145	0.333	157.7	21.7	91.0
T5—TRP 60% ^3^	0.165	0.667	179.4	43.5	92.1
Average			161.5	26.1	90.7
Stderr ^6^			2.5	0.5	0.7
*p*-value			<0.0001	<0.0001	0.041
CV, % ^7^			1.85	2.05	4.97

^1^ Basal diet; ^2^ L-Tryptophan 98%; ^3^ L-Tryptophan biomass 60%; ^4^ level of L-tryptophan (%); ^5^ L-tryptophan supplementary in the diet (g/kg); ^6^ standard error of mean; ^7^ coefficient of variation.

**Table 8:** Averages of the estimated parameters, standard errors (SEs), and fit statistics of the models, which were fitted to describe the relationship between daily egg production (%) at different daily levels of tryptophan intake supplementary (mg/hen) and the relative bioavailability value (RBV) of brown laying hens from 61 to 70 weeks of age.

Models	Parameter Estimates					RBV		
	Estimate		SE	Lower	Upper	Lower	Average	Upper
Linear AIC = 274.5 R^2^ = 91.0%	α	86.3	1.3	83.4	89.1	98.0	98.5	100.4
	βs	0.153	0.045	0.050	0.256			
	βt	0.151	0.044	0.050	0.251			
Nonlinear AIC = 273.4 R^2^ = 91.0%	α	84.8	1.5	81.5	88.2	101.0	100.0	107.0
	δ	8.3	2.3	3.0	13.5			
	βs	−0.055	0.039	−0.144	0.033			
	βt	−0.055	0.040	−0.146	0.035			

α is the intercept. The asymptote value is α + δ. βs is the slope for L-tryptophan 98%; βt is the slope for L-tryptophan biomass 60%.

## Data Availability

The data presented in this study are available on request from the corresponding author.

## Supplementary Materials

The following supporting information can be downloaded at https://www.mdpi.com/article/10.3390/ani15060866/s1. **Table S1**: Feed composition of the experimental diets (kg/ton). Table S2: Composition (kg/ton) of the basal diet used in the assay.
