# Supplementary material for: Bioavailability of Tryptophan Biomass for Laying Hens
Source: Animals (Basel). 2025 Mar 18;15(6):866. doi: 10.3390/ani15060866 (PMC11939161; doi:10.3390/ani15060866)
Supplement: Supplementary file 1 [file animals-15-00866-s001.zip › animals-3467400-supplementary.pdf]

Table S1.1 Feed composition of experimental diets (g/kg).

Ingredients	L-Tryptophan Biomass 60%	L-Tryptophan 98%
Grain Corn	715.947	715.947
Soybean Meal 46 of CP%[1]	121.925	121.925
Meat and bone meal 42 of CP%[1]	47.413	47.413
Fine Limestone	14.578	14.578
Coarse Limestone	72.146	72.146
NaCl	3.120	3.120
L-Met 100	2.823	2.823
L-Lysine	2.713	2.713
L-Threonine	1.141	1.141
L-Tryptophan	1.000	0.612
L-Valine	0.922	0.922
L-Isoleucine	1.460	1.460
L-Arginine	0.458	0.458
L-Glu 98	1.425	1.636
Starch	0.919	0.787
Choline Chloride 60% of ChoC[2]	0.710	0.710
Lignocellulose	5.150	5.305
Kaolin	5.151	5.305
Premix[3]	1.000	1.000
Total	1000	1000

[1]Crude Protein; [2]Choline Chloride; [3]Content per kg of diet: Folic Acid: 1.232 mg; Pantothenic Acid: 26.40 mg; Biotin: 0.0352 mg; Niacin: 61.60 mg; Vitamin A: 4.224 mg; Vitamin B1: 3.872 mg; Vitamin B12: 0.02112 mg; Vitamin B2: 10.032 mg; Vitamin B6: 5.632 mg; Vitamin D3: 0.1232 mg; Vitamin E: 23.584 mg; Vitamin K3: 3.52 mg; Copper: 16.00 mg; Iron: 100.00 mg; Iodine: 2.40 mg; Manganese: 140.00 mg; Selenium: 0.60 mg; Zinc: 140.00 mg.

Table S1.2 Composition (kg/ton) of the basal diet used in the assay[1].

Nutrients	**L-Tryptophan Biomass 60%**	**L-Tryptophan 98%**
DM, %	88.836	88.815
CF, %	2.198	2.198
CP, %	14.560	14.560
MEn, kcal/kg	2825	2825
Lys, %	0.746	0.746
Met, %	0.487	0.487
Met+Cys, %	0.683	0.683
Thr, %	0.525	0.525
Trp, %	0.172	0.172
Arg, %	0.777	0.777
Gly+Ser, %	1.255	1.255
Val, %	0.651	0.651
Ile, %	0.599	0.599
Leu, %	1.151	1.151
His, %	0.322	0.322
Phe, %	0.577	0.577
Phe+Tyr, %	1.018	1.018
K, %	0.478	0.478
Na, %	0.160	0.160
Cl, %	0.354	0.354
Ca, %	4.000	4.000
avP, %	0.345	0.345
Total Choline, mg/kg	1,186	1,186
Linoleic Acid, %	1,473	1,473

[1] Obtained from Rostagno, *et al.* [23]
